# Supplementary material for: The Skp1-Cullin1-FBXO1 complex is a pleiotropic regulator required for the formation of gametes and motile forms in Plasmodium berghei
Source: Nat Commun. 2023 Mar 10;14:1312. doi: 10.1038/s41467-023-36999-8 (PMC10006092; doi:10.1038/s41467-023-36999-8)

Supplementary information

Supplementary figures

Figure S1

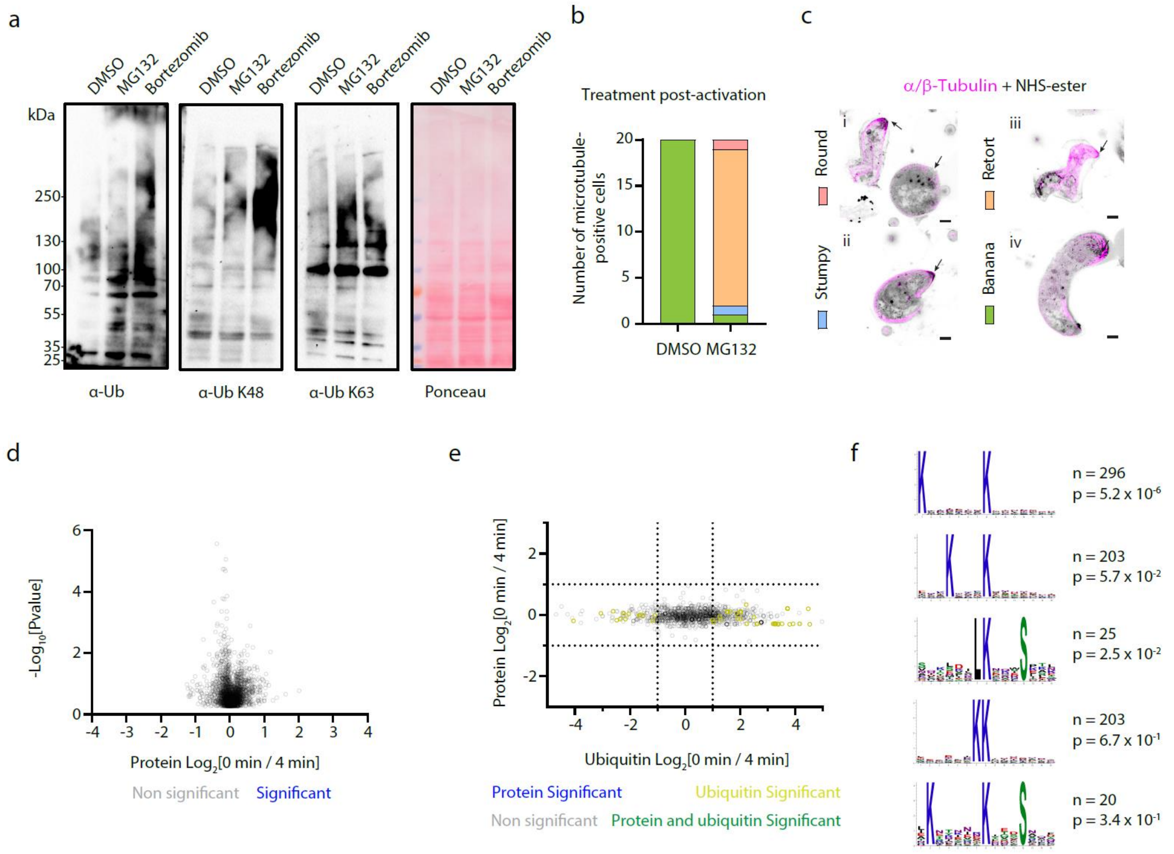

Figure S1. a. Western blot analysis with antibodies against Ubiquitin, Ubiquitin K48, and ubiquitin K63 of gametocyte lysates 10 minutes post-activation with xanthurenic acid. Gametocytes were treated pre-activation for 30 minutes with 1 µM MG132 or 25 µM bortezomib. The Ponceau staining serves as a loading control. Blots are representative of three independent replicates **b.** Treatment of gametocytes with 1 µM MG132 one hour post-activation leads to altered ookinete formation as determined by partial stack projection of expanded ookinetes (40 cells from replicate 1 of Fig 1c). **c**. Representative confocal sections showing the quantified phenotypes by U-ExM. Arrows indicate the apical pole. Scale bar = 5 µm. **d.** Volcano plot showing the extent of differentially detected proteins in non-activated and 4 min activated WT gametocytes. No significantly regulated sites (Pvalue<0.05 -

paired t-test) with a fold change >2 were identified (n = 3 biological replicates). **e.** Plot indicating relative abundance of proteins (3 biological replicates) and corresponding ubiquitinated peptides (technical duplicates from 2 biological replicates) in non-activated and 4 min activated WT gametocytes. Colours highlight significantly regulated ubiquitination events (yellow) and/ or protein levels (blue), technical duplicates from 2 biological replicates for the ubiquitome survey. **f.** Sequence logo depiction of the five significantly enriched ubiquitinated motifs detected by Motif X analysis.

Figure S2

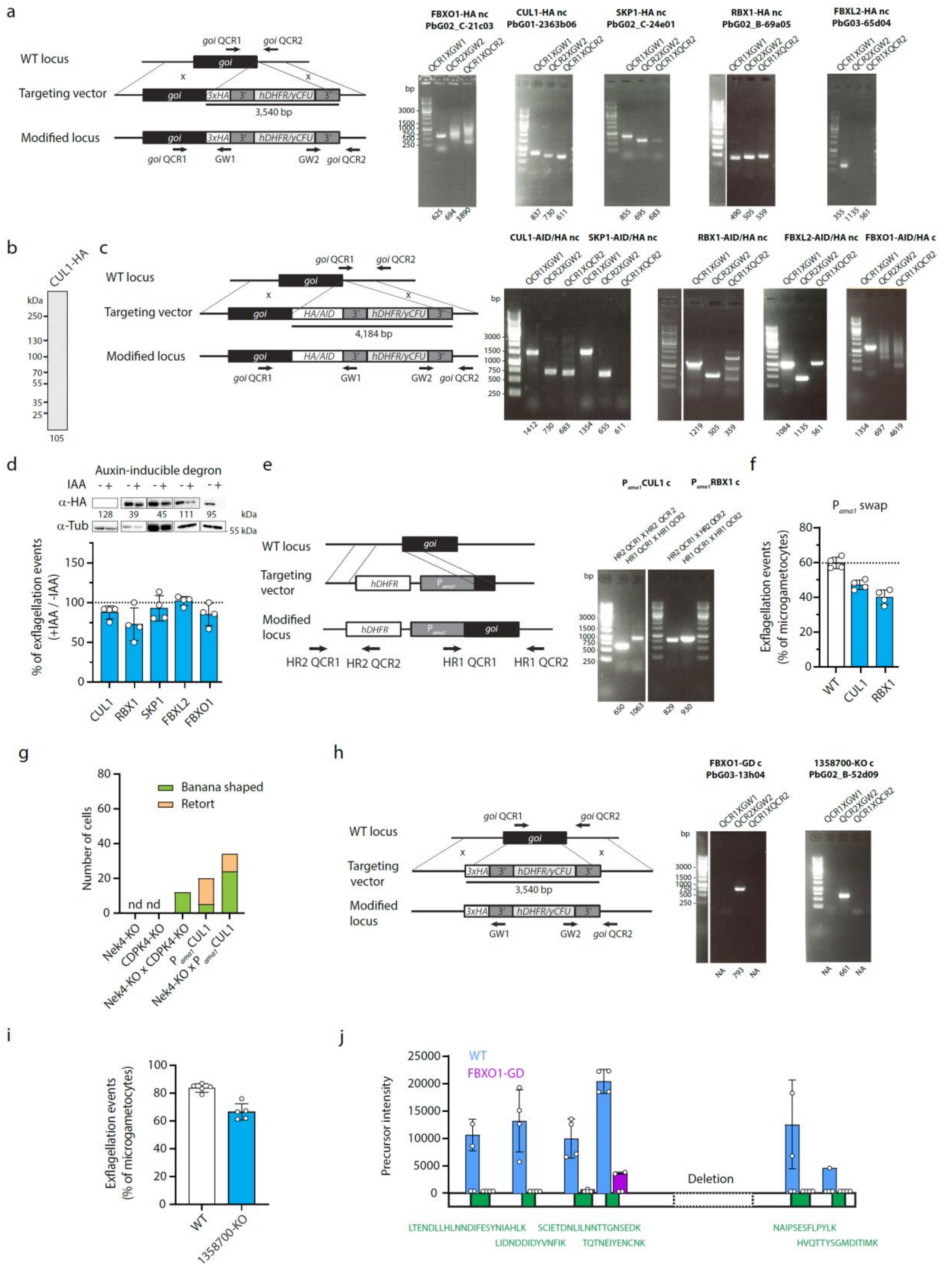

Figure S2. a. Genetic modification strategy for HA tagging and genotyping data. Oligonucleotides used for PCR genotyping are indicated and agarose gels for corresponding PCR products from genotyping

reactions are shown. The same marker size was used for all genotyping gels. PCR product sizes are indicated below each lane; NA: not amplifiable. In the line names, c is for cloned lines and nc for non-clonal lines, the ID below is the PlasmoGEM vector from which the construct was derived. **b.** Western blot analysis of a gametocyte lysate from the line expressing endogenously HA-tagged CUL1 does not allow to detect the fusion protein. The blot is representative of four independent replicates from two transgenic lines. **c.** Genetic modification strategy for AID/HA tagging and genotyping data. Oligonucleotides used for PCR genotyping of non-clonal lines (expect FBXO1-AID/HA) are indicated and agarose gels for corresponding PCR products from genotyping reactions are shown. **d.** Western blot analysis of protein degradation and relative exflagellation rate and upon IAA treatment for CUL1-AID/HA, RBX1-AID/HA, SKP1-AID/HA, FBXL2-AID/HA and FBXO1-AID/HA (error bars show standard deviation from the mean; technical replicates from four independent infections; unpaired two-tailed t-test). **e.** Genetic modification strategy for P_{ama1} promoter swaps and genotyping data. Oligonucleotides used for PCR genotyping of clonal lines are indicated and agarose gels for corresponding PCR products are shown. **f.** Effect of *cul1* and *rbx1* stage-specific downregulation on exflagellation (error bars show standard deviation from the mean; replicates from four independent infections). **g.** Number of banana-shaped or retort ookinetes from genetic crosses (66 cells counted from two biological replicates) of P_{ama1}CUL1 parasites and parasite lines defective for micro- (CDPK4-KO) and macro- (Nek4-KO) gamete formation. nd = not detected. **h.** Genetic modification strategy for gene KO or GD and genotyping data. Oligonucleotides used for PCR genotyping of clonal lines are indicated and agarose gels for corresponding PCR products are shown. **i.** Effect of *pbanka_1358700* deletion on exflagellation (error bars show standard deviation from the mean; technical replicates from four independent infections). **j.** Precursor intensities for FBXO1 in the WT (blue) and FBXO1-GD (purple) lines for detected tryptic peptides (green) – technical duplicates from two biological replicates, error bars show standard deviation from the mean.

Figure S3

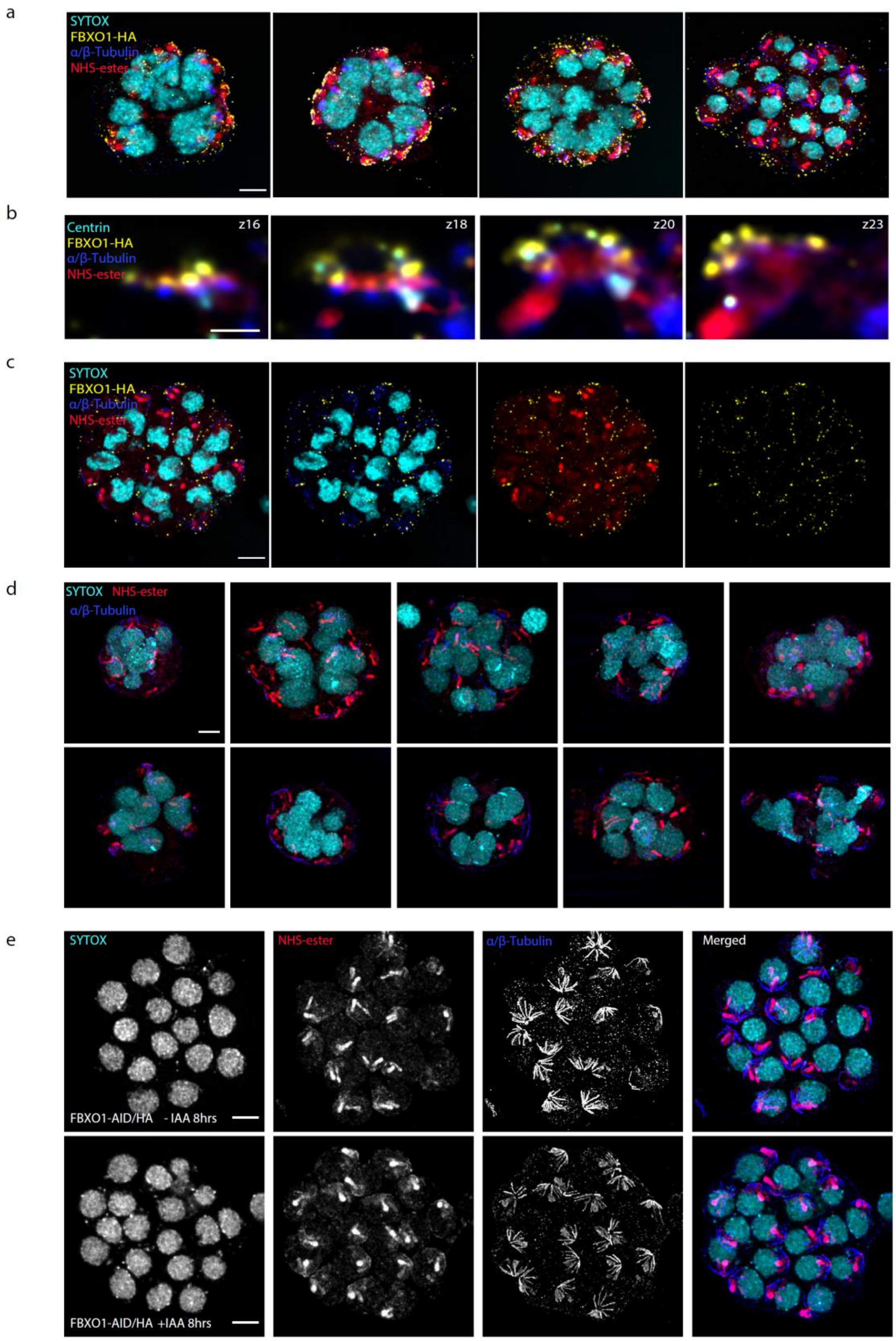

Figure S3. a. Representative full projections showing an overview of FBXO1-HA localisation during schizogony in expanded infected cells. HA: yellow; α/β-Tubulin: dark blue; amine reactive groups/NHS-

ester: red, SYTOX (DNA): cyan. Scale bars = 5 µm. **b.** Confocal sections of the boxed area shown in Fig. 3B showing the localisation of FBXO1-HA at the forming apical complex. HA: yellow; α/β-Tubulin: dark blue; amine reactive groups/NHS-ester: red, SYTOX (DNA): cyan. Scale bar = 1 µm. **c.** Representative full projections showing an overview of FBXO1-HA localisation of expanded segmented schizonts. HA: yellow; α/β-Tubulin: dark blue; amine reactive groups/NHS-ester: red, SYTOX (DNA): cyan. Scale bars = 5 µm. **d.** Gallery of full projections showing the effect of FBXO1-AID/HA depletion upon IAA/auxin addition upon parasite collection on schizogony as observed by U-ExM. α/β-Tubulin: dark blue; amine reactive groups/NHS-ester: red, SYTOX (DNA): cyan. Scale bars = 5 µm. **e.** Effect of FBXO1-AID/HA depletion upon IAA/auxin addition 8 hours post-parasite collection from mice on schizogony as observed by U-ExM. α/β-Tubulin: dark blue; amine reactive groups/NHS-ester: red, SYTOX (DNA): cyan. Scale bars = 5 µm. Images are representative of at least two independent infections.

Figure S4

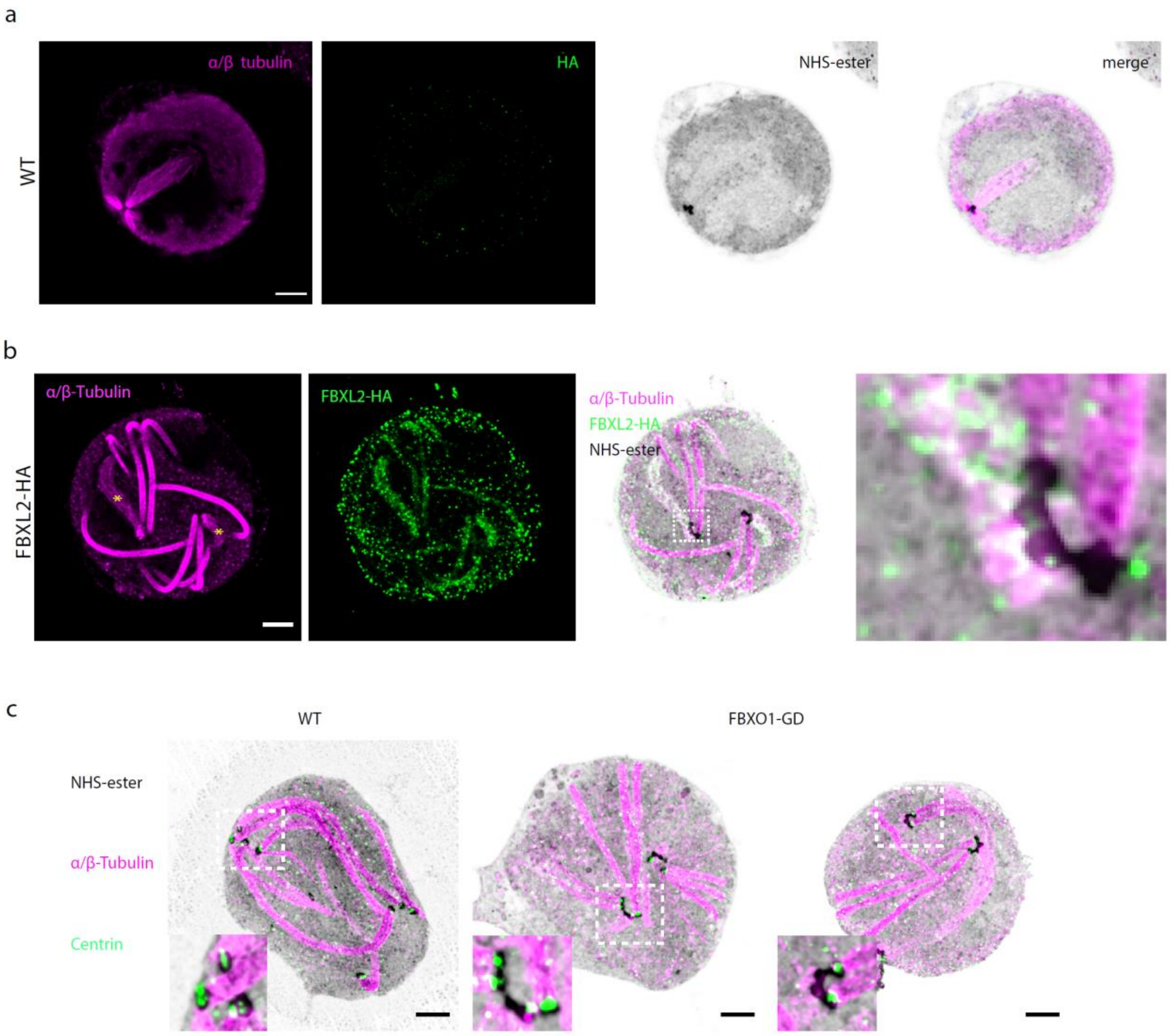

Figure S4. U-ExM characterisation of the SCF complex in gametocytes. a. Confocal section showing HA background signal after expansion in the WT line. HA: green; α/β-Tubulin: magenta; amine reactive groups/NHS-ester: shades of grey. Scale bar = 5 µm. **b.** U-ExM localisation of FBXL2-HA in activated *P. berghei* gametocytes (confocal section). HA: green; α/β-Tubulin: magenta; amine reactive groups/NHS-ester: shades of grey. The inset shows details of the region around the microtubule organisation centre. Scale bar = 5 µm. **c.** Confocal section showing segregation of microgametocyte centrosomes by U-ExM in WT and FBXO1-GD microgametocytes. Scale bar = 5 µm. Centrin: green; α/β-Tubulin: magenta; amine reactive groups/NHS-ester: shades of grey. Images are representative of two independent infections.

Figure S5

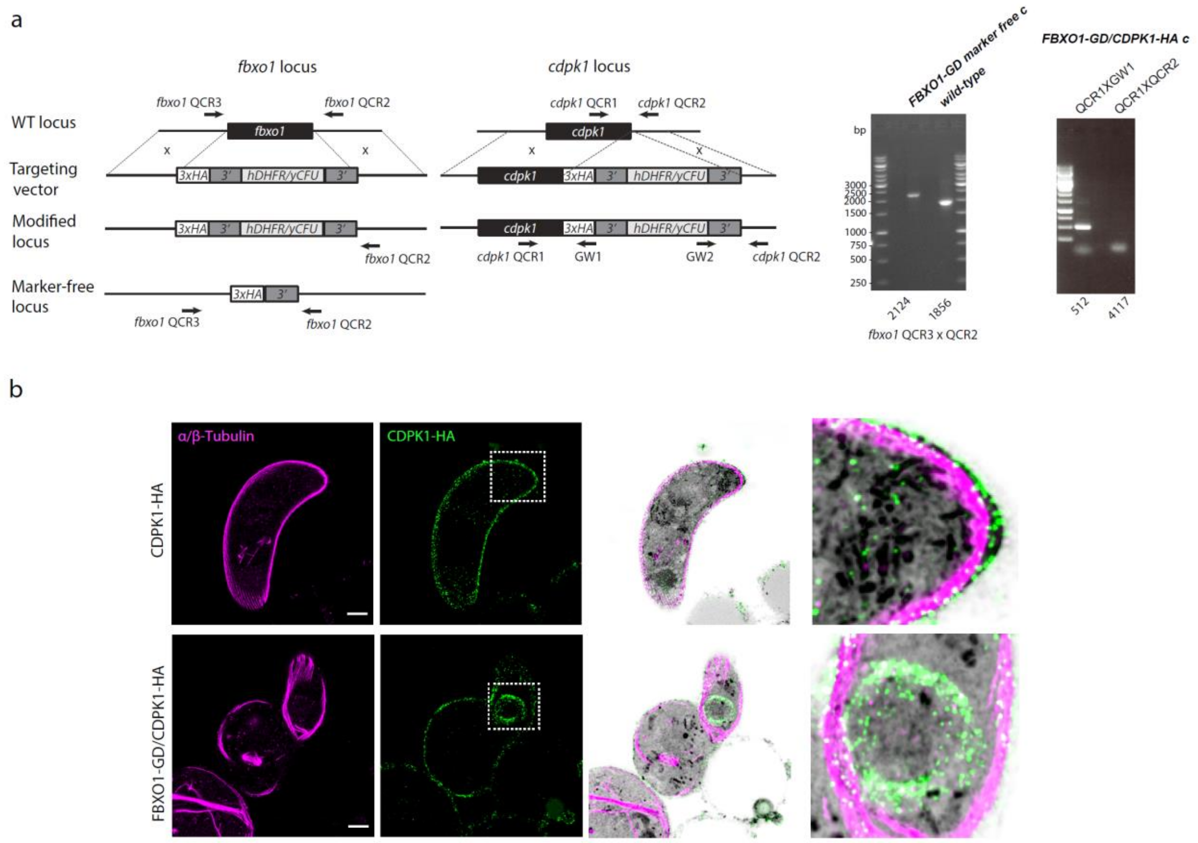

Figure S5. a. Genetic modification strategy for generating a FBXO1-GD marker-free line and a derived FBXO1-GD/CDPK1-HA clonal (c) line and genotyping data. Oligonucleotides used for PCR genotyping of clonal lines are indicated and agarose gels for corresponding PCR products from genotyping reactions are shown. **b.** Confocal section showing expanded CDPK1-HA and FBXO1-GD/CDPK1-HA ookinetes highlighting mis-localisation of CDPK1-HA upon *fbxo1* disruption. HA: green; α/β-Tubulin: magenta; amine reactive groups/NHS-ester: shades of grey. Panels on the right show details of CDPK1-HA localisation in the regions of interest. Scale bars = 5 µm. Images are representative of two independent infections.

Figure S6

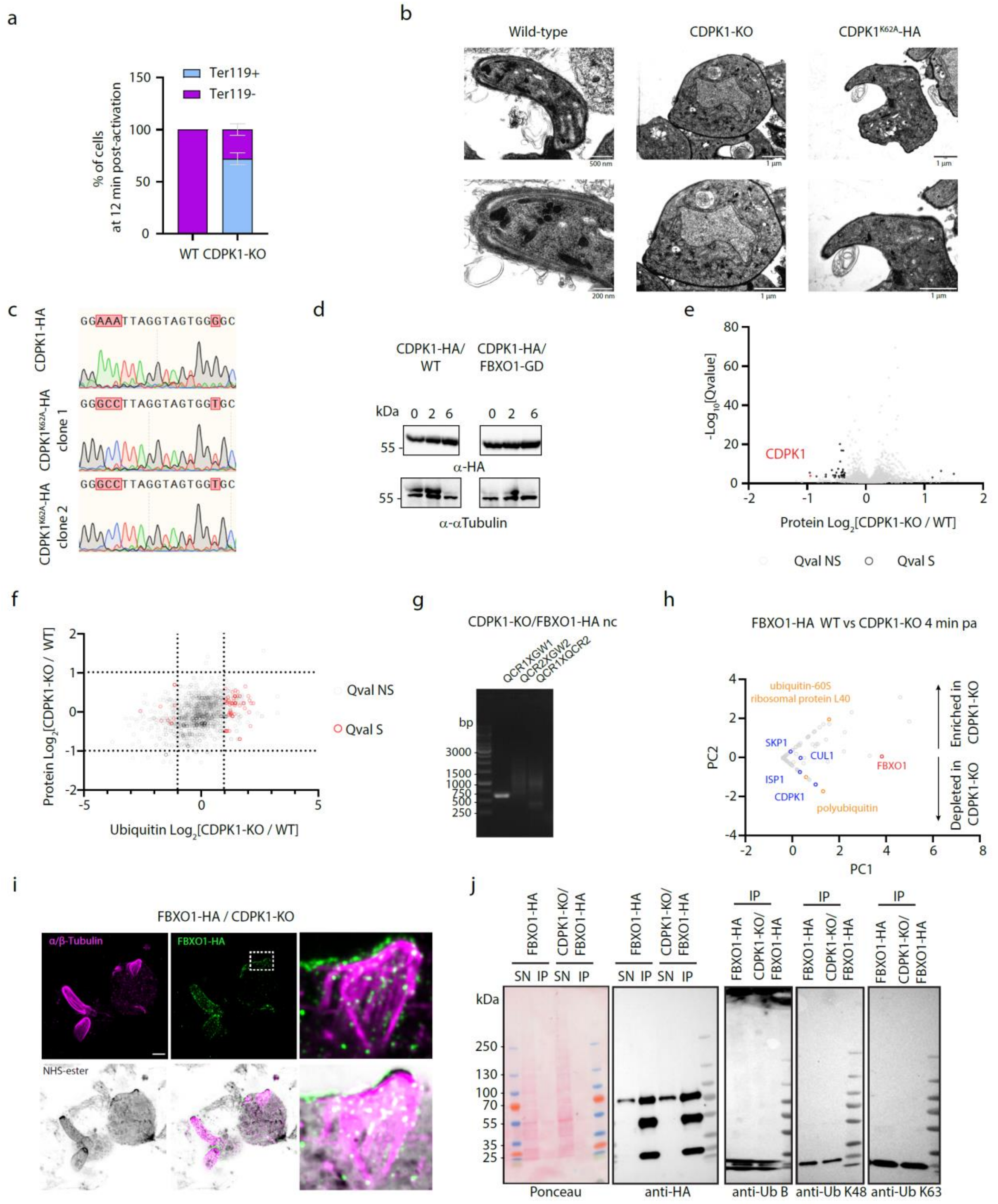

Figure S6. a. CDPK1-KO and WT gamete egress from host erythrocytes quantified by IFA based on the presence of the erythrocyte membrane marker Ter-119, 12 min post-activation (48 cells analysed from three replicates, error bars show standard deviation from the mean, one-way ANOVA). **b.** Characterisation by transmission electron microscopy of Wild-type, CDPK1-KO and CDPK1^{K62A}-HA

ookinetes 20 hours post-activation. **c.** Sequence traces showing substitution of CDPK1 lysine 62 into alanine in two independent clones in the CDPK1-HA background from reference [30]. **d.** Western blot analysis of CDPK1-HA in WT and FBXO1-GD gametocyte lysates over the course of gametogenesis. α-Tubulin serves as a loading control. Blots are representative of two independent infections. **e.** Volcano plot showing the extent of differentially detected proteins in WT and CDPK1-KO gametocytes 4 minutes post-activation. Significantly regulated sites (Qvalue<0.05 - paired t-test) with a fold change >2 are highlighted in black (n = 3 biological replicates). **f.** Plot indicating relative abundance of proteins (3 biological replicates) and corresponding ubiquitinated peptides (technical duplicates from 2 biological replicates) in CDPK1-KO compared to WT gametocytes four minutes post-activation. Red highlights significantly regulated ubiquitination events. **g.** Genotyping of FBXO1-HA tagging as in Fig. S2A in the CDPK1-KO background from reference [30]. **h.** emPAI values as identified by mass spectrometry for proteins co-purifying with FBXO1-HA, in WT and CDPK1-KO backgrounds following immunoprecipitation, and displayed in first and second principal components (n = 3 biological replicates). Red: the immunoprecipitated protein; blue: components of the SCF complex; orange: ubiquitin-related proteins differentially immunoprecipitated with FBXO1-HA in WT and CDPK1-KO backgrounds. **i.** U-ExM localisation of FBXO1-HA in CDPK1-KO ookinetes. HA: green; α/β-Tubulin: magenta; amine reactive groups/NHS-ester: shades of grey. Insets show details of the pellicle. Scale bars = 5 µm. Images are representative of two independent cultures. **j.** Western blot analysis of FBXO1-HA cell lysates and immunoprecipitates in WT and CDPK1-KO backgrounds with anti-Ub B, anti-Ub K48, and anti-Ub K63 antibodies showing no detectable ubiquitination of FBXO1-HA with these antibodies. The Ponceau staining serves as a loading control. Blots are representative of two independent infections.

Supplementary tables

Table S1. Oligonucleotides used in this study

Primer name	Sequence 5' to 3'
GW1	CATACTAGCCATTTTATGTG
GW2	CTTTGGTGACAGATACTAC
skp1 HA-F	TAATAACTCCTTTTTAGATCCGAGAAGAAAACAAATGGTGTGGAGACATTAAGGCGCATAACGATACCAC
skp1 HA-R	TTTAAAAACAAATTATAATCCTCATTTATTTAAAATAAAAAAAGGAACCTCCGCCTACTGCGACTATAGA
skp1 HA QCR1	ACGTGCATTGCAATGTTCCA
skp1 HA QCR2	ACAACTGGCATGTTTGCTAATGA
cul1 HA-F	GAGAATATATTCAGAAAGAAGAAAATAGCCAAGTATATGTTTATATACCAAAGGCGCATAACGATACCAC
cul1 HA-R	ACTCATTAGATTAGTGGCATGATAAAACGCTTCAAAATTTGGTGTCTATTCCGCCTACTGCGACTATAGA
cul1 HA QCR1	ACAATGGCCATCGAAGCAGCT
cul1 HA QCR2	TGGTCTTCACCCAAACGGCA
rbx1 HA-F	AAGTTTGCCCTTTAGATAATACTACTTGGGAATTCCAAAAAGCAACAACTAAGGCGCATAACGATACCAC
rbx1 HA-R	TAAATAAATATATATACATATTAATGATAAACACAATAATAAGAAATATTCCGCCTACTGCGACTATAGA
rbx1 HA QCR1	TGGTCAGCAGTAGCGGCATGG
rbx1 HA QCR2	ACTGGGATGGATGGCCAAAA
fbxo1 HA-F	GAGTTGGCGATCATATTGTTTTTTATTTAATTAAAGGAGGAAATAATATTAAGGCGCATAACGATACCAC
fbxo1 HA-R	GCACTGATGTAGAAATCAAGAAAAGTAGATAAGTGCATATAACATAGAGCCCGCCTACTGCGACTATAGA
fbxo1 HA QCR1	AGCTGTTGAACCGGGTAAGAGCT
fbxo1 HA QCR2	GATGCCTCTCCCCCTCCCCC
1358700 HA-F	TAAAGAGTTCTCTAAAAGAAGTTCCTTTGTGTATACACCCGACTAACCTTAAGGCGCATAACGATACCAC
1358700 HA-R	TCTATAGTTTACTTATTTACACCCCTCTTTAATTACTATATCTTAATTTTCCGCCTACTGCGACTATAGA
1358700 HA QCR1	ACCCTTCTGTTGGAGATCCTAGCCA
1358700 HA QCR2	ACCTGCCTCGAAAATACCGA
fbxl2 HA-F	AAATGTTTGAGACATCAATTTATATTGACATTGAATCCCTTGAGCAGAATAAGGCGCATAACGATACCAC
fbxl2 HA-R	TATTTTAAAGCTTCCGAAACGTGGGTATTACACTTTGCCCACATTTTTTTCCGCCTACTGCGACTATAGA
fbxl2 QCR1	TCGTTCATTTGCAGGAGCTGA
fbxl2 QCR2	TTTCTGCTCAGCCATTTCGA
cdpk1 HA QCR1	TGTCTTAGGGGAGGCTGACCA
cdpk1 HA QCR2	TCCCTGCAAATGTTTTCCGCT
fbxo1 KO-F	AAAAAAGAGTATACCCCTTATTTCAAACCAGTCAATTTTTTTTACAAATACCGCCTACTGCGACTATAGA
fbxo1 KO-R	TTATTAATTATGTGTGAAATATTTATATCATCTCCATTATTATATGATATAAGGCGCATAACGATACCAC
fbxo1 KO QCR1	CGAGACGGAAAGAGGGTTTGTCCG
fbxo1 KO QCR2	AAGAACATGTTCAATTATTTAT
fbxo1 KO QCR3	GAGTGATTTAAAATATATATTGGACTAT
1358700 KO QCR1	TGTTCTGAAAACCCCATACCT
1358700 KO QCR2	TGGCTAGGCCCATATGTGCGT
P_{ama1}*cul1* HR1 forward	TTAATAAAATAAAACGATATAAAACATGGATATATCGAGCGTTAATTTTGAAAGTGG

P_{ama1}*cul1* HR1 reverse	ATTCGCGGCCGCGATATCTCGAAATAATTTTTCATTATCTTTATCATCACTATTCCGC
P_{ama1}*cul1* HR2 forward	GCTTGACCATGATTACGCCAAGCTTGCTAATTATAAAATATTACTAGCAAGCGTCTTTTTATTCAT
P_{ama1}*cul1* HR2 reverse	AAGAATTAAGCTGGGCTGCAATTGCTCTTGATCTTTAAACTCACGTATCAG
P_{ama1}*cul1* HR1 QCR1	GGAATATAATTCAAAATGATTTGACAC
P_{ama1}*cul1* HR1 QCR2	GAGAACAATATGTAAGTTCTTCTT
P_{ama1}*cul1* HR2 QCR1	AAATATTTGTTGCAAGTAGTTA
P_{ama1}*cul1* HR2 QCR2	ATGAAAATATTACTGGTGCTTTGA
P_{ama1}*rbx1* HR1 forward	TTAATAAAATAAAACGATATAAAACTCGAGATGATTAATAATATACGATCTGAGGAAAAAGAAATATTCAAAGTTCAC
P_{ama1}*rbx1* HR1 reverse	ATTCGCGGCCGCGATATCAGAAACATAAATAAAATATTTTAACACATGAAAAAATGTCATAAAATGCG
P_{ama1}*rbx1* HR2 forward	GCTTGACCATGATTACGCCAAGCTTTATGATAGATGCTGCGTCATATAAATGATATTCCTATTTTTT
P_{ama1}*rbx1* HR2 reverse	AAGAATTAAGCTGGGCTGCAGTATAATTATTCTTCTTTCTCTTATAAAACTTATGACTTTATGAATTTATC
P_{ama1}*rbx1* HR1 QCR1	GGAATATAATTCAAAATGATTTGACAC
P_{ama1}*rbx1* HR2 QCR2	CTATTATATTATCCATTGCCAG
P_{ama1}*rbx1* HR2 QCR1	AAGTTTTCCATATTAGGGTTA
P_{ama1}*rbx1* HR2 QCR2	ATGAAAATATTACTGGTGCTTTGA
cdpk1^{K62A} HR1 forward	GCTATGACCATGATTACGCCAAGCTTAAAATAAATATACATATATGTGCGCATTTACACACACAAA
cdpk1^{K62A} HR1 reverse	TCACCATATGCACCACTACCTAAGGCCCGAACTTTAAAATACGATTCACCAAT
cdpk1^{K62A} HR2 forward	TAGGTAGTGGTGCATATGGTGAGGTTTTATTATGCAAAGAAAAGAAC
cdpk1^{K62A} HR2 reverse	TGAATATTAAATTGTAAACTTAAGGAATTCAACTTCAGGAGCTATATAATATGCAGTACCT
cdpk1^{K62A} gRNA forward	TATTAGTTCGGAAATTAGGTAGTG
cdpk1^{K62A} gRNA reverse	AAACCACTACCTAATTTCCGAACT
gRNA reverse	CAAATAGGGGTTCCGCGCAC
cdpk1^{K62A} QCR1	GTGATTTAAATTAACATGAA
cdpk1^{K62A} QCR2	ATTTCGCAAAACGTTGTATCCTTC
cdpk1^{K62A} QCR3	GAAGGATACAACGTTTTGCGAAAT
cdpk1^{K62A} QCR4	TCATTGACACGAACTCGTCGA
cdpk1^{K62A} QCR5	GTGAATAAAAAGCTATATGGTATAGC
cdpk1^{K62A} QCR6	TATATGACGGATTTCCAATTGCA
cdpk1^{K62A} QCR7	TTCCTCTCACATCATTTGCAC

Table S2. Main reagents used or generated in this study

Reagent type or resource	Designation	Source or reference	Identifiers	Additional information
Cell line *P.berghei*	ANKA 2.34 clonal	Billker *et al*, 2004		
Cell line *P.berghei*	Cullin1-AID/HA non-clonal	This study		
Cell line *P.berghei*	SKP1-AID/HA non-clonal	This study		
Cell line *P.berghei*	RBX1-AID/HA non-clonal	This study		
Cell line *P.berghei*	FBXL2-AID/HA non-clonal	This study		
Cell line *P.berghei*	FBXO1-AID/HA clonal	This study		
Cell line *P.berghei*	CUL1-HA non-clonal	This study		
Cell line *P.berghei*	SKP1-HA non-clonal	This study		
Cell line *P.berghei*	RBX1-HA non-clonal	This study		
Cell line *P.berghei*	FBXL2-HA non-clonal	This study		
Cell line *P.berghei*	FBXO1-HA marker free clonal	This study		
Cell line *P.berghei*	FBXO1-GFP non-clonal	This study		

Cell line *P.berghei*	P_{ama1}CUL1 clonal	This study		
Cell line *P.berghei*	P_{ama1}RBX1 clonal	This study		
Cell line *P.berghei*	CDPK1-HA marker free clonal	Fang *et al*, 2018		
Cell line *P.berghei*	FBXO1-GD marker free clonal	This study		
Cell line *P.berghei*	FBXO1-GD/CDPK1-HA clonal	This study		
Cell line *P.berghei*	CDPK1-KO/FBXO1-HA clonal	This study		
Cell line *P.berghei*	CDPK1-HA-K62A clone 1	This study		
Cell line *P.berghei*	CDPK1-HA-K62A clone 2	This study		
Antibody	Centrin mouse (20H5)	Merck Millipore	04-1624	U-ExM: 1:500
Antibody	α-tubulin, guinea pig	Unige antibody platform	AA345	U-ExM: 1:250
Antibody	β-tubulin, guinea pig	Unige antibody platform	AA344	U-ExM: 1:250
Antibody	MTIP	Jones *et al*, 2006		IFA: 1:1000
Antibody	HA Rat (3F10)	Roche	11815016001	U-ExM: 1:250
Antibody	GFP	Torrey Pines Biolabs	TP401	U-ExM: 1:250
Antibody	Ubiquitin (ubiquitin B)	ThermoFisher	PA1-26088	WB: 1:1000
Antibody	Ter119	Invitrogen	11-5921-82	IFA: 1:1000
Antibody	c-myc	Sigma-Aldrich	SAB4300319	WB: 1:1000
Antibody	Anti-Ubiquitin K48	Merck	ZRB2150	WB: 1:10000
Antibody	Anti-Ubiquitin K63	Merck	05-1308	WB: 1:1000
Antibody	anti-mouse Alexa 488	Invitrogen	A11001	U-ExM: 1:400
Antibody	anti-guinea pig 488	Invitrogen	A11073	U-ExM: 1:400
Antibody	anti-rat 488	Invitrogen	A11006	U-ExM: 1:400
Antibody	anti-rabbit Alexa 405	Invitrogen	A31556	U-ExM: 1:400
Antibody	anti-mouse Alexa 405	Invitrogen	A31553	U-ExM: 1:400
Antibody	anti-guinea pig Alexa 647	Invitrogen	A21450	U-ExM: 1:400
Antibody	anti-guinea pig 405	abcam	ab175678	U-ExM: 1:400
Chemical	Atto 594 NHS-ester	Merck	8741	10 µg/mL
Chemical	488 NHS-ester	ThermoFisher	46402	5 µg/ml
Chemical	Hoechst 33342	Invitrogen	H3570	IFA: 1:1000
Chemical	SYTOX™ Deep Red	ThermoFisher	S11381	U-ExM: 0.5 µM
Chemical	PTMScan® Ubiquitin Remnant Motif (K-ε-GG)	CST	5562	
Chemical	tris(2-carboxyethyl)phosphine	ThermoFisher	77720	
Chemical	Iodoacetamide	Sigma- Aldrich	I1149	
Chemical	triethylammonium bicarbonate (TEAB)	Sigma- Aldrich	T7408	
Chemical	Pierce™ 660nm Protein Assay Kit	ThermoFisher	22662	
Chemical	tris(2-carboxyethyl)phosphine	ThermoFisher	77720	

Uncropped scans of western blots shown in supplementary figures

Fig S1

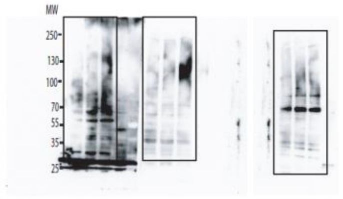

Fig S2B

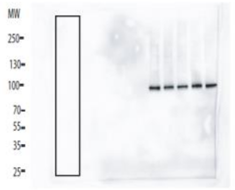

Fig S2D

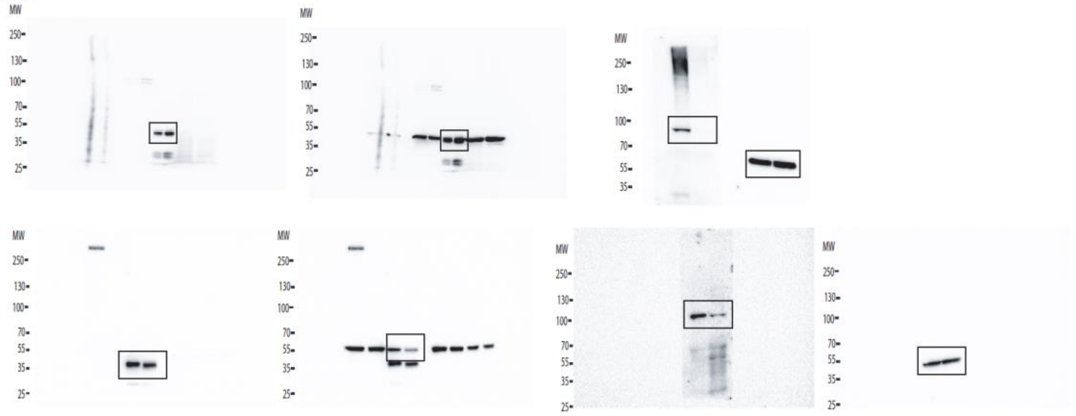

Fig S6E

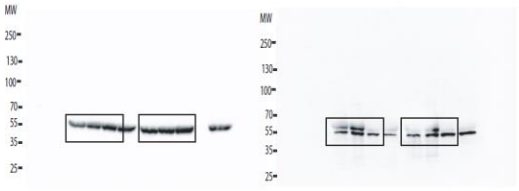

Fig S6L

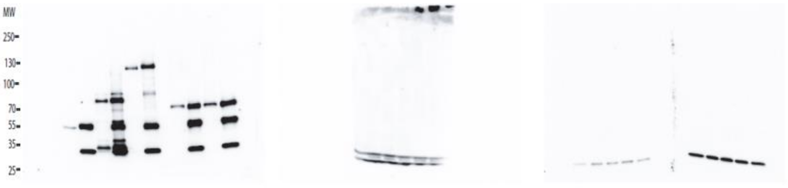

Supplement: Supplementary file 1 — Supplementary Information [file 41467_2023_36999_MOESM1_ESM.pdf]
