# Supplementary material for: The Skp1-Cullin1-FBXO1 complex is a pleiotropic regulator required for the formation of gametes and motile forms in Plasmodium berghei
Source: Nat Commun. 2023 Mar 10;14:1312. doi: 10.1038/s41467-023-36999-8 (PMC10006092; doi:10.1038/s41467-023-36999-8)
Supplement: Supplementary file 3 — Description of Additional Supplementary Files [file 41467_2023_36999_MOESM3_ESM.pdf]

Description of additional supplementary files

Title: Supplementary Data 1

Description: Proteomic and ubiquitinomic analyses in gametocytes

Title: Supplementary Data 2

Description: Proteins identified in SCFFBXO1 immunoprecipitates

Title: Supplementary Data 3

Description: Proteomic and ubiquitinomic analyses of FBXO1-GD gametocytes

Title: Supplementary Data 4

Description: Proteomic and ubiquitinomic analyses of CDPK1-KO gametocytes

Title: Supplementary Movie S1

Description: Confocal sections of expanded FBXO1-HA schizonts
