# Supplementary material for: The Skp1-Cullin1-FBXO1 complex is a pleiotropic regulator required for the formation of gametes and motile forms in Plasmodium berghei
Source: Nat Commun. 2023 Mar 10;14:1312. doi: 10.1038/s41467-023-36999-8 (PMC10006092; doi:10.1038/s41467-023-36999-8)
Supplement: Supplementary file 9 — Reporting Summary [file 41467_2023_36999_MOESM9_ESM.pdf]

nature portfolio

Corresponding author(s): Mathieu Brochet and Ravish Rashpa

Last updated by author(s): Feb 16, 2023

Reporting Summary

Nature Portfolio wishes to improve the reproducibility of the work that we publish. This form provides structure for consistency and transparency in reporting. For further information on Nature Portfolio policies, see our Editorial Policies and the Editorial Policy Checklist.

Statistics

For all statistical analyses, confirm that the following items are present in the figure legend, table legend, main text, or Methods section.

n/a	Confirmed	
☐	☒	The exact sample size (*n*) for each experimental group/condition, given as a discrete number and unit of measurement
☐	☒	A statement on whether measurements were taken from distinct samples or whether the same sample was measured repeatedly
☐	☒	The statistical test(s) used AND whether they are one- or two-sided<br>*Only common tests should be described solely by name; describe more complex techniques in the Methods section.*
☒	☐	A description of all covariates tested
☒	☐	A description of any assumptions or corrections, such as tests of normality and adjustment for multiple comparisons
☐	☒	A full description of the statistical parameters including central tendency (e.g. means) or other basic estimates (e.g. regression coefficient) AND variation (e.g. standard deviation) or associated estimates of uncertainty (e.g. confidence intervals)
☐	☒	For null hypothesis testing, the test statistic (e.g. *F*, *t*, *r*) with confidence intervals, effect sizes, degrees of freedom and *P* value noted<br>*Give P values as exact values whenever suitable.*
☒	☐	For Bayesian analysis, information on the choice of priors and Markov chain Monte Carlo settings
☒	☐	For hierarchical and complex designs, identification of the appropriate level for tests and full reporting of outcomes
☒	☐	Estimates of effect sizes (e.g. Cohen's *d*, Pearson's *r*), indicating how they were calculated

Our web collection on statistics for biologists contains articles on many of the points above.

Software and code

Policy information about availability of computer code

Data collection	iTEM FEI (Olympus), ZEN 2.6 (Zeiss) and LAS X version 3.5.7.23225 (Leica) were used for image acquisition
Data analysis	Image J 1.53, LAS X version 3.5.7.23225 (Leica) were used for image analysis<br>Excel (v1108), GraphPad Prism 9 (9.5.1) were used for data and statistical analysis<br>Bio-Rad Image Lab (6.1) was used for western blot analysis<br>Cytoscape (3.9.0), R package were used for proteomic data analysis and representation<br>ClusterProfiler 3.8 was used for Gene Ontologies term enrichment analysis<br>Motif-x algorithm proposed through the MoMo tool included in the MEME Suite was used for motif enrichment analysis<br>Spectronaut v.15 (Biognosys) was used for DIA analysis<br>Mascot (Matrix Science, London, UK; version 2.5.1) was used for the peak list files searches against the PlasmoDB_P.berghei ANKA database (PlasmoDB.org, release 38)

For manuscripts utilizing custom algorithms or software that are central to the research but not yet described in published literature, software must be made available to editors and reviewers. We strongly encourage code deposition in a community repository (e.g. GitHub). See the Nature Portfolio guidelines for submitting code & software for further information.

Data

Policy information about availability of data

All manuscripts must include a data availability statement. This statement should provide the following information, where applicable:

- Accession codes, unique identifiers, or web links for publicly available datasets
- A description of any restrictions on data availability
- For clinical datasets or third party data, please ensure that the statement adheres to our policy

All data needed to evaluate the conclusions in the paper are present in the paper and/or the Supplementary Information. Source data are provided with this paper. The mass spectrometry proteomics data generated in this study have been deposited to the ProteomeXchange Consortium via the PRIDE partner repository (http://proteomecentral.proteomexchange.org) with the dataset identifiers PXD035526 and PXD035557.

Human research participants

Policy information about studies involving human research participants and Sex and Gender in Research.

Reporting on sex and gender	NA
Population characteristics	NA
Recruitment	NA
Ethics oversight	NA

Note that full information on the approval of the study protocol must also be provided in the manuscript.

Field-specific reporting

Please select the one below that is the best fit for your research. If you are not sure, read the appropriate sections before making your selection.

[x] Life sciences [ ] Behavioural & social sciences [ ] Ecological, evolutionary & environmental sciences

For a reference copy of the document with all sections, see nature.com/documents/nr-reporting-summary-flat.pdf

Life sciences study design

All studies must disclose on these points even when the disclosure is negative.

Sample size	Sample size was determined according to the similar kind of experiments previously published by us our other groups, including for example PMID36006241, 35077503, 33705377, 32568069, 30315162 and 24594931. These sample sizes were sufficient to carry out the experiments with sufficient statistics and standard using t-test and other test.
Data exclusions	No data were excluded.
Replication	Repeated measures are shown in the Figure and legend.
Randomization	All parasite samples were randomly selected from the available population.
Blinding	The investigators were not blinded for experiments comprising objective measurements such as western blotting, IFA and U-ExM. For mass spectrometry analysis, investigators performing mass spectrometry data collection and analysis were blinded to experimental groups.

Reporting for specific materials, systems and methods

We require information from authors about some types of materials, experimental systems and methods used in many studies. Here, indicate whether each material, system or method listed is relevant to your study. If you are not sure if a list item applies to your research, read the appropriate section before selecting a response.

Materials & experimental systems

n/a	Involved in the study
☐	☒ Antibodies
☐	☒ Eukaryotic cell lines
☒	☐ Palaeontology and archaeology
☐	☒ Animals and other organisms
☒	☐ Clinical data
☒	☐ Dual use research of concern

Methods

n/a	Involved in the study
☒	☐ ChIP-seq
☒	☐ Flow cytometry
☒	☐ MRI-based neuroimaging

Antibodies

Antibodies used

Centrin, mouse (20H5), Merck Millipore,04-1624, 1:500
α-tubulin, guinea pig, Unige antibody platform, AA345, 1:250
β-tubulin, guinea pig, Unige antibody platform, AA344, 1:250
HA,Rat (3F10) Roche, 11815016001, 1:250
GFP, Torrey Pines Biolabs, TP401, 1:250
ubiquitin (ubiquitin B), ThermoFisher, PA1-26088, 1:1000
Ter119, Invitrogen, 11-5921-82, 1:1000
myc, Sigma- Aldrich, SAB4300319, 1:1000
anti-Ubiquitin K48, Merk, ZRB2150, 1:10000
anti-Ubiquitin K63, Merk, 05-1308, 1:1000
anti-mouse Alexa 488, Invitrogen, A11001, 1:400
anti-guinea pig 488, Invitrogen, A11073, 1:400
anti-rat 488, Invitrogen, A11006, 1:400
anti-rabbit Alexa 405, Invitrogen, A31556, 1:400
anti-mouse Alexa 405, Invitrogen, A31553, 1:400
anti-guinea pig Alexa 647, Invitrogen, A21450, 1:400
anti-guinea pig 405, abcam, ab175678, 1:400
anti-MTIP, PMID16513191, 1:1000

Validation

Centrin, mouse (20H5), Merck Millipore,04-1624 : Rashpa R, Brochet M (2022) PLOS Pathogens. 2022;18(1):e1010223. doi: 10.1371/journal.ppat.1010223.
α-tubulin, guinea pig, Unige antibody platform, AA345 : Rashpa R, Brochet M (2022) PLOS Pathogens. 2022;18(1):e1010223. doi: 10.1371/journal.ppat.1010223.
β-tubulin, guinea pig, Unige antibody platform, AA344 : Rashpa R, Brochet M (2022) PLOS Pathogens. 2022;18(1):e1010223. doi: 10.1371/journal.ppat.1010223.
HA,Rat (3F10) Roche, 11815016001 : Rashpa R, Brochet M (2022) PLOS Pathogens. 2022;18(1):e1010223. doi: 10.1371/journal.ppat.1010223.
GFP, Torrey Pines Biolabs, TP401 : Bertiaux E et al PLoS Biol. 2021;19(3):e3001020. doi: 10.1371/journal.pbio.3001020.
ubiquitin (ubiquitin B), ThermoFisher, PA1-26088
Ter119, Invitrogen, 11-5921-82 : Balestra AC et al Sci Adv. 2021;7(13). doi: 10.1126/sciadv.abe5396.
myc, Sigma- Aldrich, SAB4300319
Anti-Ubiquitin K48, Merk, ZRB2150 : Green JL et al PLOS Pathogens. 2020;16(6):e1008640. doi: 10.1371/journal.ppat.1008640.
Anti-Ubiquitin K63, Merk, 05-1308 : Green JL et al PLOS Pathogens. 2020;16(6):e1008640. doi: 10.1371/journal.ppat.1008640.
anti-mouse Alexa 488, Invitrogen, A11001 : Rashpa R, Brochet M (2022) PLOS Pathogens. 2022;18(1):e1010223. doi: 10.1371/journal.ppat.1010223.
anti-guinea pig 488, Invitrogen, A11073 : Rashpa R, Brochet M (2022) PLOS Pathogens. 2022;18(1):e1010223. doi: 10.1371/journal.ppat.1010223.
anti-rat 488, Invitrogen, A11006 : Rashpa R, Brochet M (2022) PLOS Pathogens. 2022;18(1):e1010223. doi: 10.1371/journal.ppat.1010223.
anti-rabbit Alexa 405, Invitrogen, A31556 : Rashpa R, Brochet M (2022) PLOS Pathogens. 2022;18(1):e1010223. doi: 10.1371/journal.ppat.1010223.
anti-mouse Alexa 405, Invitrogen, A31553 : Rashpa R, Brochet M (2022) PLOS Pathogens. 2022;18(1):e1010223. doi: 10.1371/journal.ppat.1010223.
anti-guinea pig Alexa 647, Invitrogen, A21450 : Rashpa R, Brochet M (2022) PLOS Pathogens. 2022;18(1):e1010223. doi: 10.1371/journal.ppat.1010223.
anti-guinea pig 405, abcam, ab175678 : Rashpa R, Brochet M (2022) PLOS Pathogens. 2022;18(1):e1010223. doi: 10.1371/journal.ppat.1010223.
anti-MTIP, Jones ML et al, Mol Biochem Parasitol. 2006 May;147(1):74-84. doi: 10.1016/j.molbiopara.2006.01.009.

Eukaryotic cell lines

Policy information about cell lines and Sex and Gender in Research

Cell line source(s)

The wild type P. berghei ANKA 2.34 cell line was initially obtained from Dr Oliver Billker (the Sanger Institute) from reference

	PMID15137943, the 615 cell line was obtained from Dr Nisha Philip (The University of Edinburgh) from reference PMID26118994.
Authentication	None of the cell lines were formally authenticated. Expression of Tir1-myc was checked in all 615-derived lines.
Mycoplasma contamination	Mice used to grow P. berghei were specific pathogen free (including Mycoplasma pulmonis) and subjected to regular pathogen monitoring by sentinel screening.
Commonly misidentified lines (See ICLAC register)	NA

Animals and other research organisms

Policy information about studies involving animals; ARRIVE guidelines recommended for reporting animal research, and Sex and Gender in Research

Laboratory animals	Six to twelve week-old mice were obtained from Charles River laboratories, and females were used for all experiments. Mice were specific pathogen free (including Mycoplasma pulmonis) and subjected to regular pathogen monitoring by sentinel screening. They were housed in individually ventilated cages furnished with a cardboard mouse house and Nestlet, maintained at 21 ± 2°C under a 12 hours light/dark cycle, and given commercially prepared autoclaved dry rodent diet and water ad libitum.
Wild animals	The study did not involve wild animals
Reporting on sex	Mice were solely used to grow Plasmodium berghei parasites and no analysis was performed on mice.
Field-collected samples	The study did not involve samples collected from the field
Ethics oversight	All animal experiments performed in Switzerland were conducted with the authorisation numbers GE102 and GE-58-19, according to the guidelines and regulations issued by the Swiss Federal Veterinary Office.

Note that full information on the approval of the study protocol must also be provided in the manuscript.
